# Control of Polymer Brush Morphology, Rheology, and Protein Repulsion by Hydrogen Bond Complexation

**DOI:** 10.1021/acs.langmuir.1c00271

**Published:** 2021-04-14

**Authors:** John Andersson, Gustav Ferrand-Drake del Castillo, Pierluigi Bilotto, Fredrik Höök, Markus Valtiner, Andreas Dahlin

**Affiliations:** † Department of Chemistry and Chemical Engineering, Chalmers University of Technology, 41296 Gothenburg, Sweden; ‡ Institute of Applied Physics, Group of Applied Interface Physics, Vienna University of Technology, 1040 Vienna, Austria; § Department of Physics, Chalmers University of Technology, 41296 Gothenburg, Sweden

## Abstract

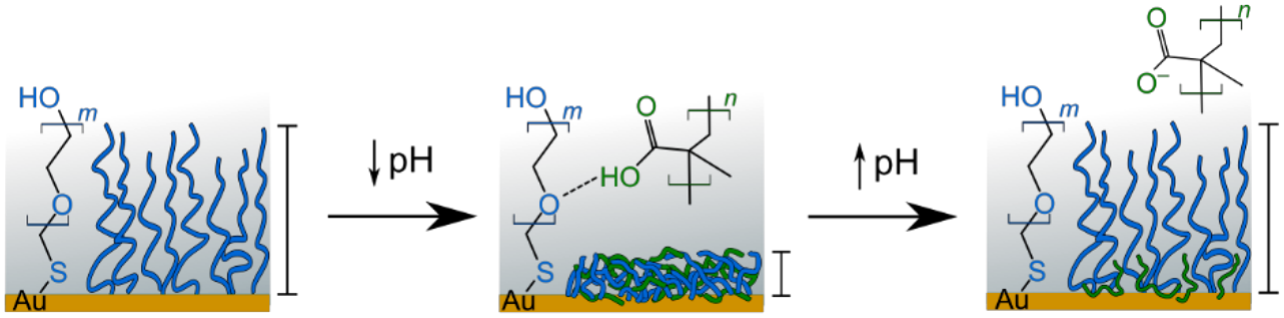

Polymer brushes are widely used to alter the properties of interfaces. In particular, poly(ethylene glycol) (PEG) and similar polymers can make surfaces inert toward biomolecular adsorption. Neutral hydrophilic brushes are normally considered to have static properties at a given temperature. As an example, PEG is not responsive to pH or ionic strength. Here we show that, by simply introducing a polymeric acid such as poly(methacrylic acid) (PMAA), the highly hydrated brush barrier can change its properties entirely. This is caused by multivalent hydrogen bonds in an extremely pH-sensitive process. Remarkably, it is sufficient to reduce the pH to 5 for complexation to occur at the interface, which is two units higher than in the corresponding bulk systems. Below this critical pH, PMAA starts to bind to PEG in large amounts (comparable to the PEG amount), causing the brush to gradually compact and dehydrate. The brush also undergoes major rheology changes, from viscoelastic to rigid. Furthermore, the protein repelling ability of PEG is lost after reaching a threshold in the amount of PMAA bound. The changes in brush properties are tunable and become more pronounced when more PMAA is bound. The initial brush state is fully recovered when releasing PMAA by returning to physiological pH. Our findings are relevant for many applications involving functional interfaces, such as capture–release of biomolecules.

## Introduction

Polymer brushes have proved important for many applications and for investigating fundamental properties of macromolecules using surface-sensitive techniques. The brush configuration is achieved when the grafting density is high enough to make the polymer coils stretch in the direction perpendicular to the surface.^1^ This can be achieved by so-called “grafting-to” approaches,^2^ where presynthesized coils are bound to the surface, or by “grafting-from” approaches,^3^ where the polymerization starts from initiators on the surface. Although a grafting-from method generally provides thicker and denser brushes, various tricks can be employed in grafting-to methods to reach fairly high grafting densities and significant chain stretching. For instance, the coils can form a covalent bond with a reactive end group under conditions where they are compacted by osmosis.^4,5^ A grafting-to method has one major advantage: the polymers can be characterized beforehand, thereby providing fundamental information such as the molecular weight distribution. The simplicity of grafting-to methods in general has led to the development of many protocols for creating brushes that prevent biomolecular adsorption or, oppositely, promote biomolecule immobilization in a gentle manner. In particular, poly(ethylene glycol) (PEG) brushes have long been used in biointerface science to make various surfaces nonfouling,^4,6^ which is critical, for instance, when it comes to interfaces in bioanalytical and biomedical devices. Lately, there has been an increasing interest in creating responsive brush interfaces that change properties depending on environmental factors.^1,7,8^ For instance, thermoresponsive polymer brushes collapse at their lower critical solution temperature^9^ and weak polyelectrolytes gradually change their degree of hydration with pH.^10^ These kinds of responsive properties make brushes interesting for various applications such as sensing, actuation, and enzyme immobilization.^11^

Molecular recognition of species introduced in solution is a relatively unexplored approach for modifying the properties of brushes that are otherwise inert and nonresponsive. Although certain brushes, in particular polyelectrolytes,^8,11,12^ are well-known for being “sticky”, there are few examples where species in solution can alter the properties of brushes that are normally repelling. In recent work, we used engineered antibodies which (through a relatively unclear interaction) can induce collapse of PEG brushes.^13^ If such specific binding events can induce fundamental changes in the brush properties, this may enable more applications and new types of responsive interfaces. Although addressed by theory^14−16^ and experimentally in a few specific cases,^13,17,18^ there are generally few examples showing how multivalent interactions (electrostatic, hydrophobic, etc.) with additives introduced in solution lead to changes in brush properties. For instance, it is established that proteins may bind to polyelectrolytes, but not how this influences brush thickness and rheology. Preferably, such changes should be investigated using well-defined chemical interactions between additives and the monomers of the grafted coils.

In this work we show how intermolecular hydrogen bonding, a phenomenon that has long been studied in the liquid bulk,^19^ can be transferred to an interface and used to drastically alter the properties of a simple hydrophilic brush. This is achieved by simply introducing a polymeric acid at low pH where it is protonated to a high extent. The PEG brush changes properties drastically due to the formation of multivalent hydrogen bonds. We quantitatively investigate brush extension, rheology changes, and protein repelling ability by a combination of surface plasmon resonance (SPR) and quartz crystal microbalance with dissipation monitoring (QCMD). Furthermore, a surface force apparatus (SFA) is used to quantify the repelling force of the PEG brush before and after hydrogen bond complexation. It is shown that the PEG brush properties are tunable based on the amount of polyacid bound and fully reversible by raising the pH. Finally, the importance of these findings for applications involving polymer brushes is discussed.

## Experimental Section

### Materials

Gold coated SPR sensors were purchased from Bionavis, and QCMD sensors were purchased from QuartzPro. ASTM research grade type 1 ultrafiltered water, referred to as MQ-water, was used for diluting all aqueous solutions. H_2_O_2_ (35%) was purchased from SAFC. NH_4_OH (25%) was purchased from Fisher Scientific. PEG (20 kg/mol, polydispersity index (PDI) 1.01) was purchased from Laysan Bio Inc. Chemicals purchased from Sigma-Aldrich include PMAA sodium salt 30 wt % solution in H_2_O (*M* = 5.4 kg/mol and PDI = 1.76), PMAA sodium salt (*M* = 75.1 kg/mol, PDI = 1.02), PMAA sodium salt (*M* = 429 kg/mol, PDI = 1.12), PMAA sodium salt (*M* = 1.2 kg/mol, PDI = 1.15), dextran from *Leuconostoc* spp. (*M* ≈ 100 kg/mol), avidin from egg white (≥98% SDS-PAGE), NaOH (anhydrous pellets), HCl (37 wt % solution), phosphate buffered saline (PBS) tablets (0.01 M Na_2_HPO_4_, 0.0027 M KCl, and 0.137 M NaCl), and Na_2_SO_4_ (≥99%).

### Preparations

Sensor surfaces were cleaned prior to PEG grafting by using an RCA1 mixture (5:1:1 volumes of MQ-water:NH_4_OH:H_2_O_2_) at 75 °C for 20 min, followed by at least three volume exchanges of MQ-water and blow-drying with N_2_. The grafting solution had 0.1 mg/mL PEG-SH in 0.2 μm filtered 0.9 M Na_2_SO_4_.^4^ Samples were incubated overnight at room temperature unless the grafting was monitored in real time. PBS buffers were prepared the same day of each experiment, degassed under vacuum in a sonication bath, and filtered with a 0.2 μm syringe filter. PBS buffers were adjusted to within 0.02 units of the target pH using 1 M HCl or NaOH after the addition of components (PMAA, dextran, or avidin) to counteract any pH change from these molecules.

### SPR Measurements and Analysis

SPR measurements were performed with a Bionavis multiparameter SPR Navi 220A instrument equipped with 670, 785, and 980 nm laser diodes. Data in figures is shown for 670 nm. The backside of each sensor was cleaned by rubbing with lens tissue soaked in 2-propanol followed by blow-drying of both sides with N_2_. All SPR measurements were performed at a set temperature of 25 °C at a flow rate of 20 μL/min for measurements in liquid (except for the PEG grafting solution at 2 μL/min). At least two repeats of each measurement in air were performed for all samples to verify no significant signal drift occurred due to adsorption of moisture. Dry thickness was determined by fitting Fresnel models implemented in MATLAB as described previously.^10−12^ After PMAA binding, samples were rinsed with MQ-water containing only a small amount of HCl to lower the pH and to avoid salt crystals on the surface. No significant difference in thickness (<1 nm) was obtained when modeling PMAA as either above or underneath the PEG layer. For calculating exclusion heights, thickness and refractive index values for the metal layers were determined separately by using a reference chip treated identically but not exposed to PEG (see examples in our previous work^10,20^). Fresnel modeling was performed on averaged reflectivity spectra selected shortly before and after injection of dextran (example in Figure S3).

### QCMD Measurements and Analysis

QMCD measurements were performed with a Q-Sense E4 instrument (Biolin Scientific) and a NE-1000 syringe pump (New Era pump systems) for flow control. All measurements were performed at a set temperature of 25 °C at a flow rate of 100 μL/min. Voight modeling^21^ and curve fitting was performed with the instrument specific software package Qtools using overtones 3, 5, 7, and 9. To obtain frequency and dissipation signals relative to a blank QCMD crystal, the absolute frequency and dissipation were recorded in the same liquid environment prior to PEG grafting.

### SFA Measurements and Analysis

A new prototype SFA including a semiconductor strain gauge was used for simultaneous measurements of interaction forces and absolute distance.^22^ Experiments were conducted in a clean room environment within laminar flow hoods (ISO class 1). Physical vapor deposition was used to deposit 2 nm of Cr and 30 nm of Au on the substrate for the polymer brush (glass disk with 2 cm radius of curvature). The PEG brush was prepared on the freshly deposited Au film by using the same procedure as on SPR and QCMD sensor surfaces. The substrate was inserted into the SFA cell and probed against a freshly cleaved back-silvered mica surface glued on a glass disk with the same radius of curvature.

## Results and Discussion

Inspired by previous work on hydrogen bond interactions with polyacidic brushes,^10,12,20^ we investigated how a PEG brush responded when exposed to polymeric acids at different pHs using SPR and QCMD. Throughout this paper, we present results for 20 kg/mol PEG brushes grafted directly to gold by specific end-group thiol binding. In brief, the grafting density is increased by shrinking the PEG coils by osmosis using Na_2_SO_4_.^4^ The resulting PEG brushes follow the de Gennes scaling law and have proved to be highly protein repelling.^4^ Furthermore, we show results for poly(methacrylic acid) (PMAA) introduced in solution, but we emphasize that many other polymer combinations (one as brush and one in solution) are possible as long as one has carboxylic acid groups.^12,19,23^ The PEG brushes have the advantage of measurable grafting density (0.25 ± 0.05 nm^–2^ depending on the exact batch of thiol-PEG and incubation time) since the molecular weight is known. The grafting density is measured on each sample by SPR spectra obtained in the dry state,^24^ after which further analysis is done by real-time measurements in liquid. Also, using PEG enables us to compare our results with the established PEG–PMAA interaction in solution,^25−28^ where 1:1 monomer complexation occurs under highly acidic conditions.^23^

**Figure 1:**
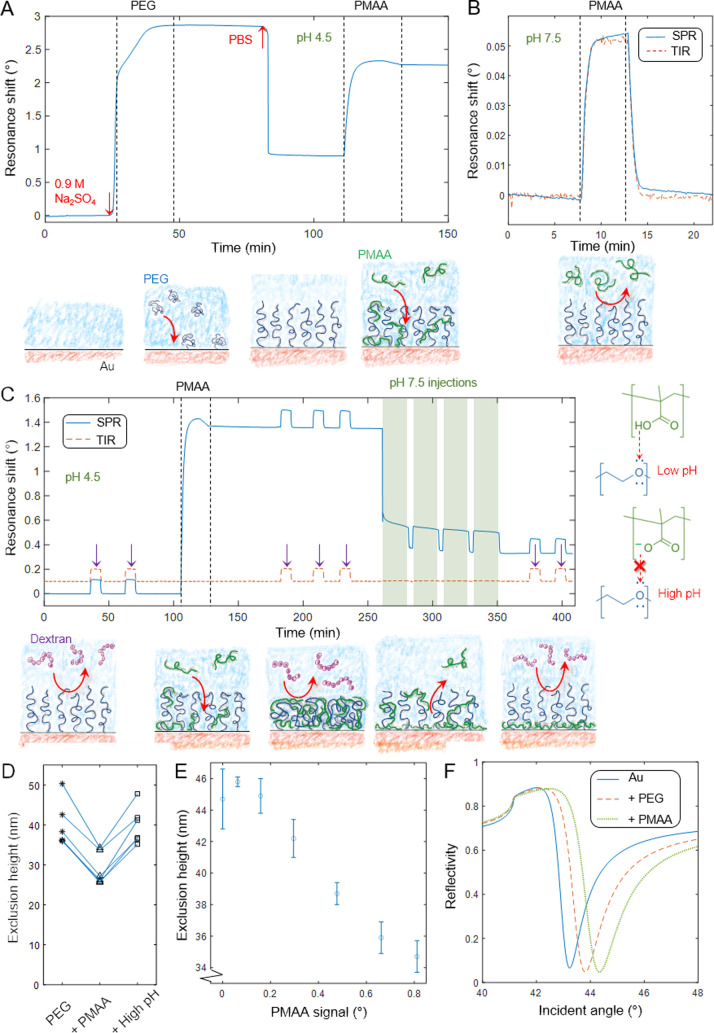
Analyzing intermolecular complex formation by SPR. (A) Kinetics of 20 kg/mol thiol-PEG grafting in 0.9 M Na_2_SO_4_ and subsequent 8 kg/mol PMAA binding (100 μg/mL) in PBS with pH lowered to 4.5. (B) Control shows only a bulk response when a high concentration (10 mg/mL) of PMAA is introduced at physiological pH. (C) Kinetics of PMAA (100 μg/mL introduced) binding to and dissociating from a PEG brush as the pH is changed. The arrows indicate dextran injections to probe brush height. (D) Exclusion heights of the PEG brush initially, after saturated PMAA binding, and after rinsing at high pH. Each line represents one experiment. (E) Example of exclusion height as a function of amount of PMAA bound at pH 4.5. (F) Angular spectra in dry state of clean Au, Au + PEG brush, and after saturated PMAA binding.

As a first observation, we noted that the hydrogen bond interactions were extremely pH sensitive: PMAA (∼8 kg/mol or more) did not bind at all until reaching a critical pH of pH_crit_ 5.2 ± 0.1, after which high amounts (comparable to the PEG amount) bound very quickly with lower pH. As soon as binding was clearly observed, it was also found to be irreversible; i.e., it was very difficult to identify conditions where the PMAA spontaneously dissociated from the PEG brush. The sharp contrast in binding behavior occurred in a pH interval much more narrow (less than one unit) than for other pH-responsive brushes and hydrogels, which tend to give continuous changes over a much broader pH range.^10,20,29^ The pH-sensitive nature of the hydrogen bond complexation is illustrated by the high SPR signal at pH 4.5 (Figure 1), while there was no detectable binding above pH_crit_. For instance, at pH 7.5, even injections of very high PMAA concentrations (10 mg/mL) gave responses which perfectly followed the total internal reflection (TIR) angle (Figure 1B), confirming that the refractive index only increased in the liquid bulk.^10,13^ The sharp transition in binding behavior with pH suggests that a certain fraction of the carboxylic acid groups need to be protonated to act as hydrogen bond donors.^19^ Indeed, when using a much smaller PMAA (1.3 kg/mol), which has fewer potential hydrogen bond donors in total, the pH_crit_ was lower (around 4.5). Our interpretation is that practically all monomers need to be protonated for such a short chain to bind. The PMAA polydispersity limits accurate quantitative analysis of the number of hydrogen bonds required for attachment. Nevertheless, on the basis of a theoretical model for degree of protonation,^30^ we estimated the number of hydrogen bonds required for PMAA attachment to lie between 10 and 50 (Supporting Information). Interestingly, previously pH_crit_ ≈3 has been measured for PEG–PMAA complexation in bulk.^26,28^ This is at least two units lower than what we observed (Figure S1), which can be partly attributed to the fact that the interaction occurs at an interface. The surface confinement of the PEG chains enforces a high local monomer concentration, which is expected to result in a stronger affinity for PMAA. Tentatively, this is largely due to favorable entropic contributions from multiple bond configurations, which become especially important for flexible molecules such as polymers.^31^ Regardless, the remarkably increased pH_crit_ is beneficial as it opens up for utilizing the hydrogen bond complexation in milder environments. For instance, most proteins will retain their structure at pH 5 but not at pH 3.^32^

Note that the binding kinetics of PMAA to the PEG brush showed a small but consistent “overshoot”; i.e., the association curve has a maximum (Figure 1A). This is characteristic for multivalent interactions and occurs even though the bulk concentration remains constant^13^ (as verified by the TIR angle). We attribute this behavior to initial weak attachment of many chains with relatively few hydrogen bonds and long “dangling ends”. As equilibrium is established, some chains desorb from the brush while others attach strongly by forming more bonds. Similar effects have been observed when polymers adsorb to solid surfaces.^33,34^

In previous work, we have shown that full angular SPR spectra can be used to probe brush heights using the bulk signal from noninteracting macromolecular probes and Fresnel models.^9−12^ In brief, the thicker the brush, the smaller the response from the probe due to the quickly decaying evanescent field.^17^ The result from this method is an “exclusion height”, representing the characteristic distance from the surface at which the probe is prevented from entering further into the brush. Note that this probes the height of the *hydrated* brush, and by comparing with the dry thickness (by SPR spectra in air), the degree of hydration can be estimated. In this work we used dextran as probe (Figure 1C) because it does not interact significantly with either PEG or PMAA.^12^ This was confirmed by an almost fully linear relation between the SPR and TIR angles during injections (Figure S3). As a control, we verified that the exclusion height of the PEG brush was independent of pH (Figure S4). The initial height had some sample-to-sample variation influenced by PEG batch, surface reuse, etc. Regardless of the initial value, we consistently observed a decrease of 12 ± 2 nm upon PMAA binding (Figure 1D). Additionally, since PMAA remains bound to the brush below pH_crit_, we could probe the brush height with different amounts of PMAA on the surface (Figure 1E). The results show a gradual decrease of the exclusion height; i.e., the system acts as a soft nanoscale actuator. It may seem counterintuitive that the addition of material to a thin film makes it even thinner, but this behavior has been observed for brushes both theoretically^14^ and experimentally^12,13^ if their degree of hydration is high. Based on the dry thickness and the exclusion height, our PEG brushes have a water volume fraction of at least 80% before PMAA binding, although it should be kept in mind that this is an average across the parabolic density profile.^4,13,24^

A fraction of the bound PMAA could not be removed by repeated rinsing with increased pH (Figure 1C). We attribute this to primary adsorption of PMAA to the gold surface underneath the PEG brush (see also Figure S5 and related discussion). The brush is an impenetrable barrier to most molecules that would readily adsorb to gold (e.g., proteins in general^4,24^), but PMAA, which clearly interacts with PEG (at low pH), is able to “slip through” the brush and reach the solid surface. The exclusion height as a function of amount of bound PMAA did not show a decrease initially (Figure 1E), which could be because PMAA first fills up the underlying gold. Interestingly, the primary adsorbed PMAA did not have any significant effect on any of the brush properties investigated in this study; i.e., after desorbing (most of) the PMAA by raising pH, the brush behaved the same as before it was exposed to PMAA. For instance, the exclusion height was fully recovered (Figure 1D). A small SPR signal appears in response to pH changes (Figure 1C), but this is expected simply due to changes in ionization state of the remaining PMAA.^10,35^

In order to determine the stoichiometry of the PEG–PMAA complex, we compared the signals from the polymers. Although absolute quantification of SPR signals can be complicated in liquid, a relative comparison is straightforward: The SPR signals are 0.90° from PEG grafting and 1.37° from PMAA binding (Figure 1A). Taking into account the differences in mass-based refractivity^4,10,35^ (0.134 and 0.158 cm^3^/g for PEG and protonated PMAA, respectively, Figure S6) and monomer weight (44 and 86 g/mol), the resulting stoichiometry is 1:0.66 (EG:MAA). Further analysis was done by Fresnel models of angular spectra measured in the dry state^10−12^ (Figure 1E), assuming dry refractive index values of 1.456 for PEG^4^ and 1.522 for PMAA.^12^ The ratio of the fitted thicknesses was converted to mass coverage using the densities (1.09 g/cm^3^ for PEG^4^ and 1.22 g/cm^3^ for PMAA^12^) and then to a molar ratio using the monomer weights. This yielded a comparable stochiometric ratio of 1:0.75. Note that both estimates ignore that some PMAA (∼20% based on the remaining signal after pH increase) is interacting with the gold surface and not with PEG. Furthermore, there could be a certain number of free dangling ends of immobilized PMAA chains. Hence, the amount of PMAA is considerably lower than what is expected from the 1:1 equilibrium ratio observed for bulk complexation.^23^ However, we observed that when further lowering pH to about 4 (the limit due to PMAA precipitation in PBS buffer) the saturated SPR signal more than doubled, which means there are more MAA than EG monomers. Hence, it appears that the hydrogen bonds, which will become more numerous (per PMAA chain) at lower pH, are competing with other effects such as increased entropic penalties from deeper insertion into the PEG brush.^18^

**Figure 2:**
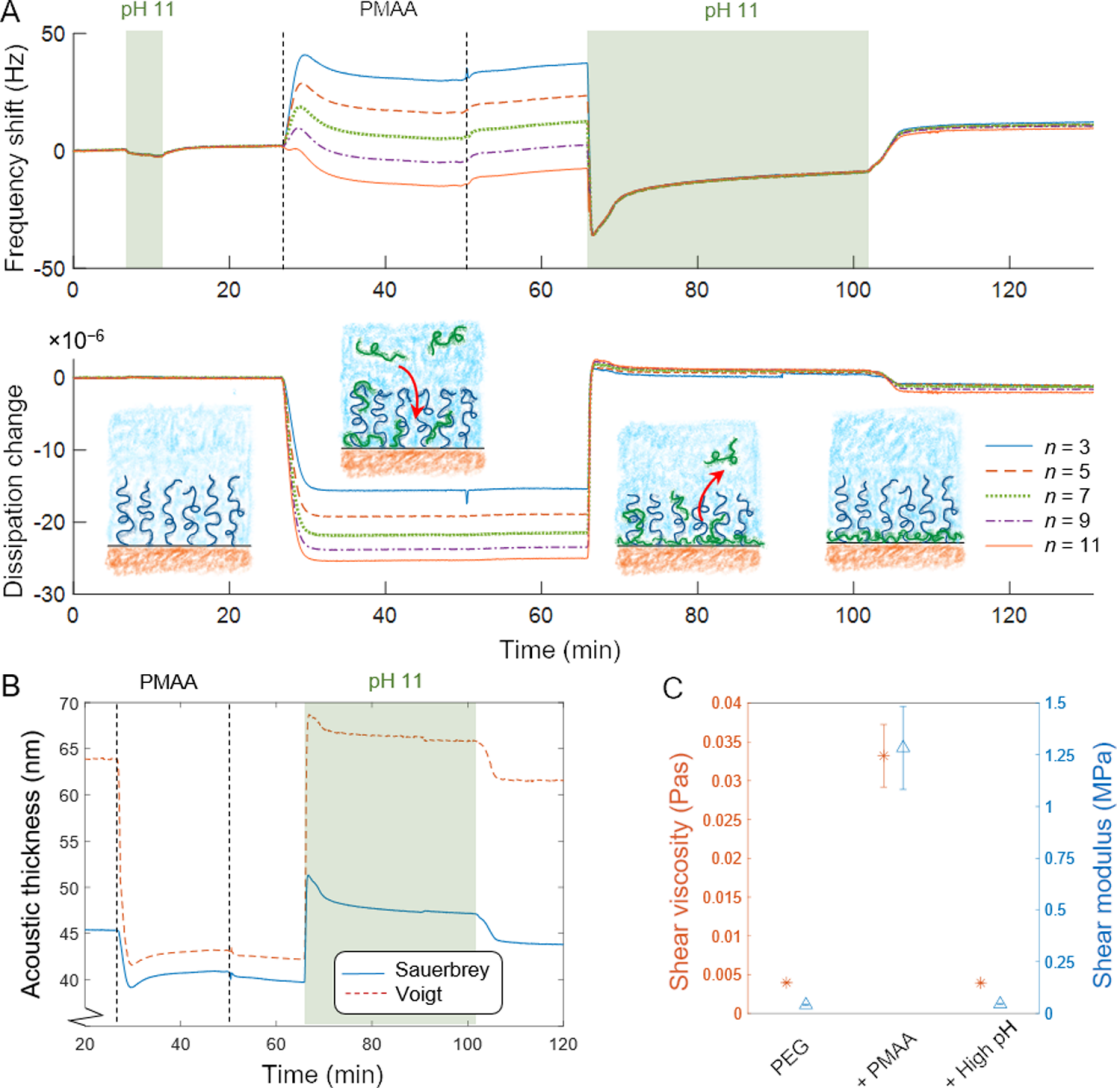
Probing rheology changes in the brush by QCMD. (A) Frequency and dissipation signals at different overtones upon PMAA (100 μg/mL) binding at pH 4.5 and release by increased pH. (B) Thickness changes upon PMAA binding based on Voight and Sauerbrey models. (C) Change in shear elasticity and viscosity upon complexation. After a pH increase the initial values are recovered.

To obtain information about rheology changes in the brush, the interaction was characterized by QCMD.^36^ As PMAA bound to PEG, a complex frequency response was observed with strong dependence on overtone number *n* (Figure 2). Notably, at *n* = 3, the end frequency shift was *positive*, which shows that the water expulsion from the brush dominates over the added PMAA mass.^9^ The response decreased in magnitude and became negative at *n* = 9 or more. The strong dependence on overtone suggests major changes in viscoelastic properties of the film. In order to model these, we also measured the reference frequency and dissipation of the bare sensor crystal in PBS.^36^ This showed that the signals divided by respective overtone number actually become identical *after* PMAA binding (see alternative plot in Figure S7). Thus, the PEG brush is initially viscoelastic and rigidifies after hydrogen bond complexation. This is further supported by the large decrease in the dissipation, which also reached low values in absolute numbers after complexation (<3 × 10^–6^ compared to the bare crystal at all overtones). After the pH was raised, minor changes in frequency (∼10 Hz) and dissipation (∼1 × 10^–6^) were observed compared to the initial values (Figure 2A). This is consistent with some remaining PMAA directly adsorbed to gold, which does not strongly influence the viscoelastic response of the brush above.

Figure 2B shows the calculated brush heights during the PMAA binding and subsequent pH increase, using either the Sauerbrey constant (0.057 cm^2^ ng^–1^ Hz^–1^) at the third overtone or multiparameter Voight modeling^21^ (fits in Figure S8). In both cases, the film density was set to 1.1 g/cm^3^, which is the average of PEG, PMAA, and water. (This value must be quite accurate since all components have similar densities.) In the initial brush state, the Voight thickness is 64 nm (Figure 2B), i.e., considerably higher than the exclusion heights measured by SPR (Figure 1D). The value is, however, well below the average contour length of the chains^4,24^ (∼130 nm). It should be kept in mind that the brush–solution boundary is highly dynamic and not well-defined due to the parabolic density profile.^4^ Thermal fluctuations will make a fraction of the chains extend more than the average height at any point in time. Furthermore, although the PEG chains have low polydispersity (1.01), some will still be considerably longer than the average. In the end, the acoustic thickness has a different physical meaning than the exclusion height and should be more sensitive to a few chains extending a bit further. Previous work on polymer films has shown similar behavior when Voight-based heights are compared with those obtained from optical methods.^37,38^ The initial Sauerbrey height is actually in better agreement with the exclusion height, even though the brush is highly hydrated.^36^ However, this is not surprising because our data fulfill the requirement that Δ*D*/Δ*f* < 4 × 10^–7^ Hz^–1^ (Figure S7), which is commonly used as a rule of thumb for when the Sauerbrey constant (17.7 ng/cm^2^ Hz^–1^) can give an accurate thickness under the assumption that the film density is correct.^36^ (In other words, even if the accurate “dry” surface mass coverage cannot be obtained, the film thickness can still be extracted.)

Upon PMAA binding, the Voight height decreases considerably to ∼42 nm, while the Sauerbrey height only decreases down to about 40 nm. The Voight height thus shows a higher change in thickness compared to SPR, while the Sauerbrey model shows a much smaller change. Both models give a good agreement with the SPR height after complexation, but Sauerbrey is again closer. However, viscoelastic models that account for the variation in response with overtone number^21,39^ are needed to obtain information about the complex modulus of the brush. Figure 2C shows the elastic shear modulus and viscosity for the pure PEG brush, after PMAA binding and after rinsing with increased pH. The initial viscosity is 4 × 10^–3^ Pa·s, which is in good agreement with that for a diluted PEG solution.^40^ Upon PMAA complexation, the elastic modulus changes by orders of magnitude from 50 kPa to 1.3 MPa, which is comparable to a rubber band. The viscosity increases almost an order of magnitude to ∼0.03 Pa·s, similar to a 40% (by weight) PEG solution.^40^ To the best of our knowledge, this is the first study showing how a hydrophilic polymer brush undergoes quite large rheology changes due to molecular binding by multivalent interactions. Previously, hydrogen bond complexation on surfaces has only been studied by using other types of thin film coatings.^29,41−43^ There are also some similarities to the recently reported strong change in friction between polyelectrolyte brushes caused by multivalent cations.^44^

**Figure 3:**
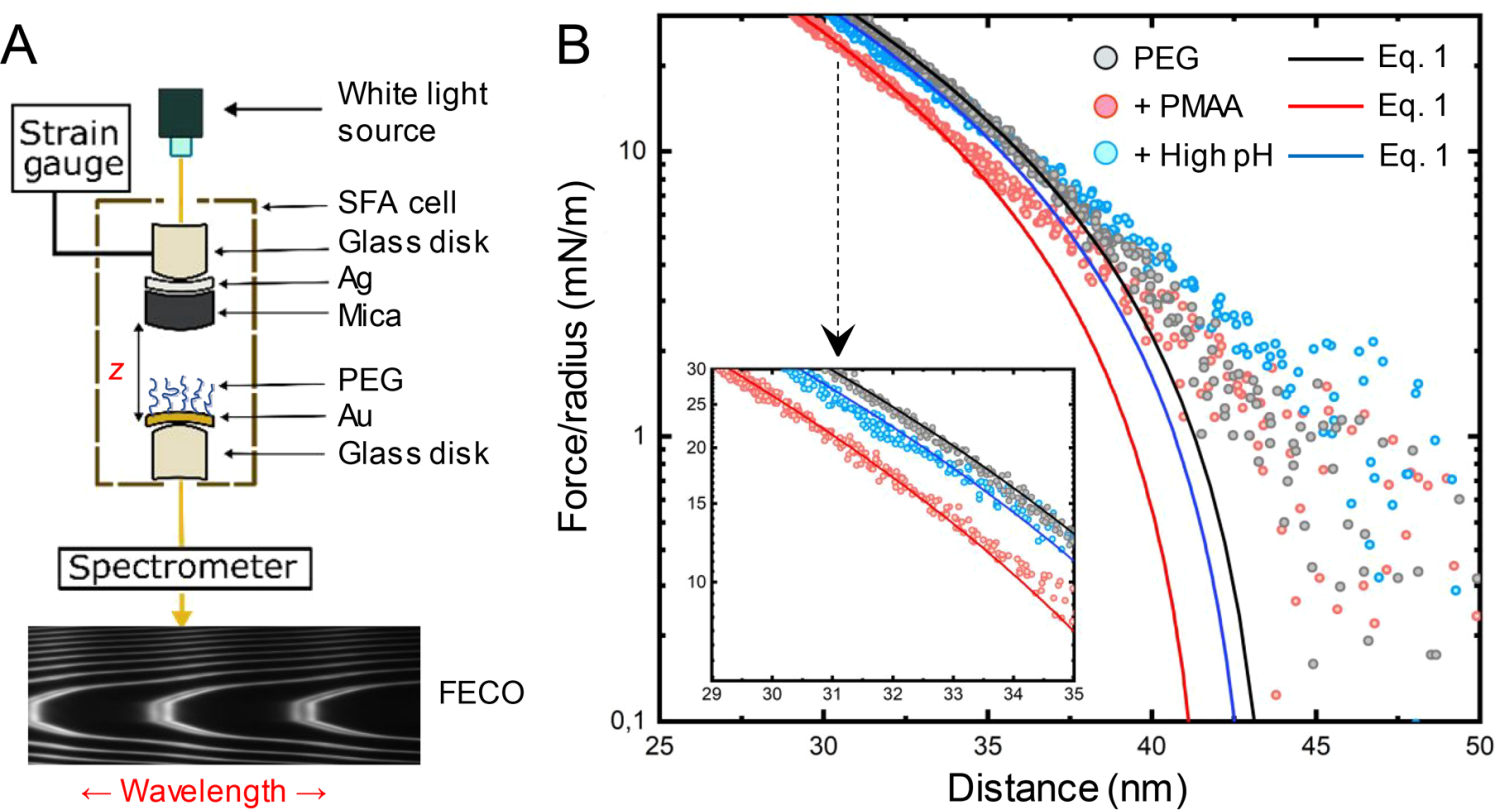
Surface force apparatus experiments. (A) Simplified representation of the setup. The PEG brush is approached by a mica surface at a decreasing distance *z* and the force is measured. The separation between the surfaces is measured optically based on multiple beam interference fringes of equal chromatic order^48^ (FECO). (B) Semilogarithm plot of the force–distance curve profile during the approach of a mica surface to a PEG brush in pH 4.5 PBS (black markers), after PMAA bound to the PEG brush (red markers), and after increase of pH to 7.5 PBS (blue markers). The full lines are fits to eq 1, resulting in equilibrium brush heights (no compression) of 44.0, 42.0, and 43.4 nm. The inset shows the behavior of the polymer brush in the compressed regime (linear in the semilog plot).

In order to verify the results from SPR and QCMD, we used an SFA^22,45^ to further analyze the PEG brush changes upon complexation. There are some important differences between SFA measurements and brush compression by an atomic force microscopy (AFM) probe:^13,24^ First, the absolute distance to the approaching surface is directly measured with an interferometric method, rather than using the relative *z*-movement from the piezo. Second, in SFA the approaching surface is compressing the whole brush, while a sharp AFM tip would penetrate it.^4^ We performed force–distance experiments with the PEG brush on gold facing a clean mica surface (Figure 3). First, we analyzed surface contact in air in order to estimate the dry PEG thickness, resulting in a value of 9.75 nm (Figure S9), which is similar to the higher values obtained from SPR spectra in air (up to 9.3 nm). The small discrepancy is likely due to the roughness of the gold film. Afterward, force profiles of the solvated PEG brush were recorded in PBS with a pH adjusted to pH 4.5. Next, we exposed the PEG brush to PMAA solution for 30 min and rinsed with PBS (still at pH 4.5), followed by another measurement. Finally, we increased the pH to 7.5 and recorded a further set of force versus distance data points. The results are presented as a semilog plot in Figure 3B.

The force curves in aqueous environment featured rather similar shapes, indicative of a brush compression, although with notable differences. The repelling force from the brush compression starting at the equilibrium height *H* can be described by a de Gennes model:^45^
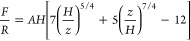
1

Here *z* < *H* for the case of brush compression. The terms containing *z* represent conformational entropy favoring compression and osmotic pressure favoring swelling. *R* is the radius of the SFA disk, and the additional term containing a factor 12 comes from integrating the original de Gennes expression.^46^ Also, the Derjaguin approximation is used for two cylinders in the limit *z* ≪ *R*.^47^ In our asymmetric geometry of a polymer brush facing a solid surface, we define the prefactor *A* as in the work by Kuhl et al.^45^ but a factor of 2 lower since we compress only one brush:
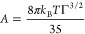
2

Here Γ is the grafting density as molecules per area which we determined by SPR measurements in the dry state^4^ (0.25–0.3 nm^–2^). After some compression (*z*/*H* < 0.9), all force–distance curves are expected to approach an exponential dependence,^47^ in good agreement with the data (inset in Figure 3B).

We fitted eq 1 to the initial PEG brush compression in the range 35 < *z* < 50 nm, allowing both *A* and *H* to vary^45^ (Figure 3B). This gave a brush thickness of *H* = 44 nm, in very good agreement with the SPR exclusion height. Furthermore, we fitted *A* = 4.4 × 10^5^ N/m^2^, which indeed lies within the range predicted by eq 2 ((3.7–4.6) × 10^5^ N/m^2^) based on the experimental variation in Γ. Next, we consider the force–distance data with PMAA bound. It is evident that this curve is significantly shifted compared to the other two. Equation 1 is not necessarily expected to work well in this case because the layer is closer to a stiff hydrogel than a brush (consider the QCMD results). Nevertheless, we still performed a fit and allowed only *H* to vary (keeping the prefactor fixed to 4.4 × 10^5^ N/m^2^). The fit was still fair (*R*^2^ = 0.987) and resulted in a small but significant reduction in brush height to 42 nm. Although this decrease is smaller than the change in height measured by SPR (Figure 1D) and QCMD (Figure 2B), there is qualitative agreement. Treating the film as a Hookean solid led to a much poorer fit (not shown). Finally, we performed a fit for the compression of the same PEG brush after raising the pH. This led to an almost full recovery of the initial value of *H* (Figure 3B). Thus, the SFA results confirm that the remaining primary adsorbed PMAA has no significant effect on the height or the repelling properties of the brush. Note that it is highly unlikely that PMAA is causing degrafting of the PEG chains since all methods (SPR, QCMD, and SFA) confirm that the brush regains its initial properties after the pH is increased.

A summary of all brush heights obtained by the different methods is given in Table 1. Clearly, there is fair agreement for the methods, even though they probe the brush height in very different ways. The most notable deviation is the QCMD Voight model, which predicts a thicker PEG brush before PMAA complexation. Also, the SPR exclusion height undergoes stronger changes than the acoustic (QCMD) and contact (SFA) heights. This is likely illustrating that the latter two methods are more sensitive to a few chains (PEG or PMAA) extending a bit further from the surface than the rest.

**Table 1:** Summary of Brush Heights Obtained by Different Methods

method	PEG brush	after PMAA binding	after raising pH	comments
SPR exclusion height	40 nm	28 nm	40 nm	similar to previously reported value (38 nm) with protein probes^4^
QCMD Sauerbrey (third overtone)	45 nm	40 nm	44 nm	assumes rigid film
QCMD Voight	64 nm	43 nm	62 nm	fully models viscoelastic properties
SFA	44 nm	42 nm	43 nm	by fitting to de Gennes model
AFM	42 nm	not measured	not measured	from previous report^4^

**Figure 4:**
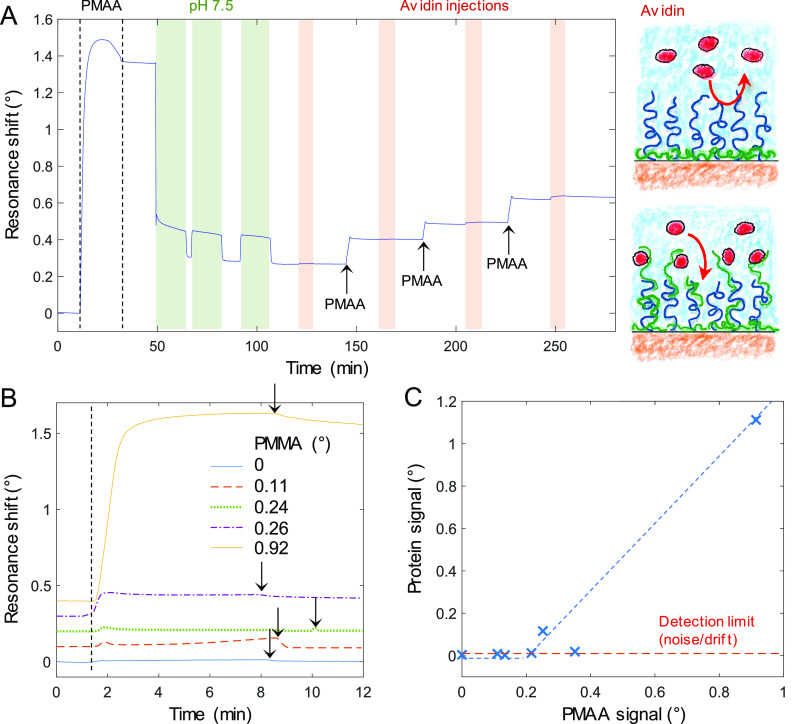
Altering the protein repelling ability of PEG brushes. (A) PMAA binding followed by rinsing steps at pH 7.5 and injection of avidin, which gives no detectable binding. Subsequently, very brief injections of PMAA and new injections of avidin are performed. (B) Avidin injections for different amounts of PMAA already bound to the PEG brush (represented by SPR signals). Rinsing is performed as indicated by arrows. (C) Avidin signal as a function of PMAA signal. The detection limit is indicated. The dashed lines are guides to the eye, showing a threshold behavior.

Finally, we investigated how the protein repelling ability of the PEG brush was altered upon hydrogen bond complexation. In recent work, we showed that PMAA also hydrogen bonds very efficiently with proteins in its protonated state.^12^ This raises the question of whether the PEG brush still repels proteins after complexation with PMAA. In order to find out, we tested if a model protein (avidin) would adsorb to the brush. An example is shown in Figure 4, which first shows once more the PMAA binding and subsequent rinsing steps by high pH. Afterward, upon the first injection of avidin, no binding is detected, confirming that the protein repelling ability of the PEG brush is not influenced by the remaining PMAA. This further strengthens the view of PEG brushes as strong kinetic barriers preventing protein adsorption.^24,49^ If a protein finds its way down to the gold surface, it should adsorb, but this does not occur (at least not on the time scale of the experiment). Next, we performed very brief injections of PMAA to bind only a small amount at a time and measured the response from avidin after each step (Figure 4A). Examples of responses from avidin injections with different amounts of PMAA bound are shown in Figure 4B.

Figure 4C summarizes the irreversible signals from avidin (after rinsing) as a function of the signal from PMAA, excluding the signal corresponding to the amount adsorbed directly to gold. A threshold behavior is observed where a certain amount of PMAA (corresponding to ∼0.2°) needs to be bound before the protein starts to bind. This threshold value is similar to the PMAA signal at which the exclusion height starts to decrease significantly (Figure 1E), further supporting that primary adsorption occurs first. Eventually, when PMAA is surely binding to PEG, fairly high amounts of protein can be immobilized. The highest signal of 1.1° corresponds to at least one dense protein monolayer.^4,11^ When the pH was raised, all proteins were desorbed together with the PMAA that bound to PEG as expected. These results suggest that, at low coverages, very few protonated PMAA monomers are available to interact with the proteins. As the amount of PMAA increases, more and longer free dangling ends will appear, enabling hydrogen bonds to form with proteins as well. In other words, the PMAA acts as a bridge between PEG and proteins, similar to layer-by-layer assembly of elastomeric films.^43^ Qualitatively, we observed the same behavior with QCMD and also for other proteins such as bovine serum albumin. However, some large proteins such as β-glucuronidase appeared to “extract” PMAA from the brush instead of binding to it (negative signals were observed). This suggests that the PMAA–protein interactions can sometimes outcompete the intermolecular polymer complexation, which is not surprising since the binding strength to the protein depends on its molecular weight and surface groups.^12^ Larger proteins also impose a higher entropic penalty upon insertion into the brush.^18^

## Conclusion

This work shows that an ordinary PEG brush, a construct which is widely used to prevent biomolecular adsorption both *in vitro* and *in vivo*, can alter its properties drastically in the presence of polyacids at low pH due to the formation of multivalent hydrogen bonds. Importantly, it is sufficient to go down to a pH of about 5, not 3 as in bulk systems used to study the same interactions. Using multiple techniques, we have shown that the brush reduces its degree of hydration and decreases in thickness as a function of the amount of PMAA bound. Furthermore, the brush becomes much more rigid, as expected when the chains become locked in a certain configuration due to the multivalent interaction. In addition, the protein repelling properties are lost after a certain threshold in the amount of bound PMAA is reached. The process can in some ways be regarded as a form of physical gelation, yet the long PMAA chains are not that similar to cross-link connection points. All changes in brush properties are fully reversible with pH; i.e., after going back to physiological pH, the PEG brush exhibits its usual barrier properties once more.

This study is the first to demonstrate how interactions with solute molecules in the form of hydrogen bond complexation influence the properties of a PEG brush. We believe the results are important since PEG brushes are widely used for making surfaces “nonfouling”. Our study provides awareness of the fact that they will alter their properties entirely in the presence of neutral PMAA. Note that our system differs from the use of “mixed brushes” containing multiple polymers that may hydrogen bond.^8^ Such brushes are tunable by changing bulk liquid parameters, not by introducing macromolecular species in solution while maintaining the same physicochemical environment. As the protonated weak polyacids also interact with many other hydrophilic polymers,^19^ the results are important for many other brush systems as well. Indeed, we are currently investigating the hydrogen bond complexation with polymer brushes prepared by grafting-from methods. Several applications are possible based on the hydrogen bond complexation. For instance, one can envision the capture and subsequent release of molecules carrying a recognition “tag” in the form of a PMAA chain. The target molecules could potentially even be of biological origin, such as proteins with engineered sequences of repeated aspartic acid or glutamic acid residues.^50^

## Supporting Information Available

Additional QCMD data and model fitting, estimation of number of hydrogen bonds for attachment, example SPR data for probing brush height, molar refractivity of protonated PMAA, supplementary SFA data (PDF)

## References

[ref1] Chen W.-L.; Cordero R.; Tran H.; Ober C. K. 50th anniversary perspective: Polymer brushes: Novel surfaces for future materials. Macromolecules 2017, 50, 4089–4113. 10.1021/acs.macromol.7b00450.

[ref2] Zdyrko B.; Luzinov I. Polymer brushes by the ″grafting to″ method. Macromol. Rapid Commun. 2011, 32, 859–869. 10.1002/marc.201100162. 21509848

[ref3] Zoppe J. O.; Ataman N. C.; Mocny P.; Wang J.; Moraes J.; Klok H. A. Surface-initiated controlled radical polymerization: state-of-the-art, opportunities, and challenges in surface and interface engineering with polymer brushes. Chem. Rev. 2017, 117, 1105–1318. 10.1021/acs.chemrev.6b00314. 28135076

[ref4] Emilsson G.; Schoch R. L.; Feuz L.; Hook F.; Lim R. Y. H.; Dahlin A. B. Strongly stretched protein resistant poly(ethylene glycol) brushes prepared by grafting-to. ACS Appl. Mater. Interfaces 2015, 7, 7505–7515. 10.1021/acsami.5b01590. 25812004

[ref5] Ortiz R.; Olsen S.; Thormann E. Salt-induced control of the grafting density in poly(ethylene glycol) brush layers by a grafting-to approach. Langmuir 2018, 34, 4455–4464. 10.1021/acs.langmuir.8b00030. 29583002

[ref6] Gidi Y.; Bayram S.; Ablenas C. J.; Blum A. S.; Cosa G. Efficient one-step PEG-silane passivation of glass surfaces for single-molecule fluorescence studies. ACS Appl. Mater. Interfaces 2018, 10, 39505–39511. 10.1021/acsami.8b15796. 30346695

[ref7] Stuart M. A. C.; Huck W. T. S.; Genzer J.; Muller M.; Ober C.; Stamm M.; Sukhorukov G. B.; Szleifer I.; Tsukruk V. V.; Urban M.; Winnik F.; Zauscher S.; Luzinov I.; Minko S. Emerging applications of stimuli-responsive polymer materials. Nat. Mater. 2010, 9, 101–113. 10.1038/nmat2614. 20094081

[ref8] Bratek-Skicki A.; Cristaudo V.; Savocco J.; Nootens S.; Morsomme P.; Delcorte A.; Dupont-Gillain C. Mixed polymer brushes for the selective capture and release of proteins. Biomacromolecules 2019, 20, 778–789. 10.1021/acs.biomac.8b01353. 30605604

[ref9] Emilsson G.; Schoch R. L.; Oertle P.; Xiong K.; Lim R. Y. H.; Dahlin A. B. Surface plasmon resonance methodology for monitoring polymerization kinetics and morphology changes of brushes - evaluated with poly(N-isopropylacrylamide). Appl. Surf. Sci. 2017, 396, 384–392. 10.1016/j.apsusc.2016.10.165.

[ref10] Ferrand-Drake del Castillo G.; Emilsson G.; Dahlin A. Quantitative analysis of thickness and pH actuation of weak polyelectrolyte brushes. J. Phys. Chem. C 2018, 122, 27516–27527. 10.1021/acs.jpcc.8b09171.

[ref11] Ferrand-Drake del Castillo G.; Koenig M.; Muller M.; Eichhorn K.-J.; Stamm M.; Uhlmann P.; Dahlin A. Enzyme immobilization in polyelectrolyte brushes: High loading and enhanced activity compared to monolayers. Langmuir 2019, 35, 3479–3489. 10.1021/acs.langmuir.9b00056. 30742441

[ref12] Ferrand-Drake del Castillo G.; Hailes R. L. N.; Adali-Kaya Z.; Robson T.; Dahlin A. Generic high-capacity protein capture and release by pH control. Chem. Commun. 2020, 56, 5889–5892. 10.1039/D0CC01250E. 32373823

[ref13] Emilsson G.; Sakiyama Y.; Malekian B.; Xiong K.; Adali-Kaya Z.; Lim R. Y. H.; Dahlin A. B. Gating protein transport in solid state nanopores by single molecule recognition. ACS Cent. Sci. 2018, 4, 1007–1014. 10.1021/acscentsci.8b00268. 30159397 PMC6107858

[ref14] Gu C.; Coalson R. D.; Jasnow D.; Zilman A. Free energy of nanoparticle binding to multivalent polymeric substrates. J. Phys. Chem. B 2017, 121, 6425–6435. 10.1021/acs.jpcb.7b00868. 28631928

[ref15] de Beer S.; Mensink L. I. S.; Kieviet B. D. Geometry-dependent insertion forces on particles in swollen polymer brushes. Macromolecules 2016, 49, 1070–1078. 10.1021/acs.macromol.5b01960.

[ref16] Kim J. U.; O’Shaughnessy B. Morphology selection of nanoparticle dispersions by polymer media. Phys. Rev. Lett. 2002, 89, 238301 10.1103/PhysRevLett.89.238301. 12485045

[ref17] Schoch R. L.; Lim R. Y. H. Non-interacting molecules as innate structural probes in surface plasmon resonance. Langmuir 2013, 29, 4068–4076. 10.1021/la3049289. 23437874

[ref18] Schneck E.; Berts I.; Halperin A.; Daillant J.; Fragneto G. Neutron reflectometry from poly (ethylene-glycol) brushes binding anti-PEG antibodies: Evidence of ternary adsorption. Biomaterials 2015, 46, 95–104. 10.1016/j.biomaterials.2014.12.041. 25678119

[ref19] Hydrogen-Bonded Interpolymer Complexes: Formation, Structure and Applications; Khutoryanskiy V. V., Staikos G., Eds.; World Scientific: Hackensack, NJ, 2009; pp x, 366.

[ref20] Ferrand-Drake del Castillo G.; Hailes R. L. N.; Dahlin A. Large changes in protonation of weak polyelectrolyte brushes with salt concentration - implications for protein immobilization. J. Phys. Chem. Lett. 2020, 11, 5212–5218. 10.1021/acs.jpclett.0c01289. 32515599 PMC7467743

[ref21] Voinova M. V.; Rodahl M.; Jonson M.; Kasemo B. Viscoelastic acoustic response of layered polymer films at fluid-solid interfaces: continuum mechanics approach. Phys. Scr. 1999, 59, 391–396. 10.1238/Physica.Regular.059a00391.

[ref22] Bilotto P.; Lengauer M.; Andersson J.; Ramach U.; Mears L. L. E.; Valtiner M. Interaction profiles and stability of rigid and polymer-tethered lipid bilayer models at highly charged and highly adhesive contacts. Langmuir 2019, 35, 15552–15563. 10.1021/acs.langmuir.9b01942. 31475831

[ref23] Osada Y.; Sato M. Thermal equilibrium of intermacromolecular complexes of polycarboxylic acids realized by cooperative hydrogen-bonding. J. Polym. Sci., Polym. Lett. Ed. 1976, 14, 129–134. 10.1002/pol.1976.130140302.

[ref24] Emilsson G.; Xiong K.; Sakiyama Y.; Malekian B.; Ahlberg Gagner V.; Schoch R. L.; Lim R. Y. H.; Dahlin A. B. Polymer brushes inside solid state nanopores form an impenetrable entropic barrier for proteins. Nanoscale 2018, 10, 4663–4669. 10.1039/C7NR09432A. 29468241

[ref25] Zeghal M.; Auvray L. Structure of polymer complexes in water. Europhys. Lett. 1999, 45, 482–487. 10.1209/epl/i1999-00192-1.

[ref26] Ikawa T.; Abe K.; Honda K.; Tsuchida E. Interpolymer complex between poly(ethylene oxide) and poly(carboxylic acid). J. Polym. Sci., Polym. Chem. Ed. 1975, 13, 1505–1514. 10.1002/pol.1975.170130703.

[ref27] Tsuchida E.; Osada Y.; Ohno H. Formation of interpolymer complexes. J. Macromol. Sci., Part B: Phys. 1980, B17, 683–714. 10.1080/00222348008212832.

[ref28] Ohno H.; Matsuda H.; Tsuchida E. Aggregation of poly(methacrylic acid)-poly(ethylene oxide) complex in aqueous medium. Makromol. Chem. 1981, 182, 2267–2275. 10.1002/macp.1981.021820816.

[ref29] Sudre G.; Hourdet D.; Creton C.; Cousin F.; Tran Y. Probing pH-responsive interactions between polymer brushes and hydrogels by neutron reflectivity. Langmuir 2014, 30, 9700–9706. 10.1021/la501568p. 25099624

[ref30] Arnold R. The titration of polymeric acids. J. Colloid Sci. 1957, 12, 549–556. 10.1016/0095-8522(57)90060-0.

[ref31] Martinez-Veracoechea F. J.; Leunissen M. E. The entropic impact of tethering, multivalency and dynamic recruitment in systems with specific binding groups. Soft Matter 2013, 9, 3213–3219. 10.1039/c3sm27766f.

[ref32] Konermann L. Protein unfolding and denaturants. In eLS; John Wiley & Sons Ltd.: Chichester, U.K., 2012; 7 pp. 10.1002/9780470015902.a0003004.pub2.

[ref33] Dorgan J. R.; Stamm M.; Toprakcioglu C. Adsorption kinetics of end-attaching triblock copolymers. Polymer 1993, 34, 1554–1557. 10.1016/0032-3861(93)90882-B.

[ref34] Leermakers F. A. M.; Gast A. P. Block copolymer adsorption studied by dynamic scanning angle reflectometry. Macromolecules 1991, 24, 718–730. 10.1021/ma00003a015.

[ref35] Currie E. P. K.; Sieval A. B.; Avena M.; Zuilhof H.; Sudhölter E. J. R.; Cohen Stuart M. A. Weak polyacid brushes: Preparation by LB deposition and optically detected titrations. Langmuir 1999, 15, 7116–7118. 10.1021/la990689p.

[ref36] Reviakine I.; Johannsmann D.; Richter R. P. Hearing what you cannot see and visualizing what you hear: Interpreting quartz crystal microbalance data from solvated interfaces. Anal. Chem. 2011, 83, 8838–8848. 10.1021/ac201778h. 21939220

[ref37] Saftics A.; Prosz G. A.; Turk B.; Peter B.; Kurunczi S.; Horvath R. In situ viscoelastic properties and chain conformations of heavily hydrated carboxymethyl dextran layers: a comparative study using OWLS and QCM-I chips coated with waveguide material. Sci. Rep. 2018, 8, 11840 10.1038/s41598-018-30201-6. 30087383 PMC6081421

[ref38] Eisele N. B.; Labokha A. A.; Frey S.; Gorlich D.; Richter R. P. Cohesiveness tunes assembly and morphology of FG nucleoporin domain meshworks-Implications for nuclear pore permeability. Biophys. J. 2013, 105, 1860–1870. 10.1016/j.bpj.2013.09.006. 24138862 PMC3797576

[ref39] Domack A.; Prucker O.; Ruhe J.; Johannsmann D. Swelling of a polymer brush probed with a quartz crystal resonator. Phys. Rev. E: Stat. Phys., Plasmas, Fluids, Relat. Interdiscip. Top. 1997, 56, 680–689. 10.1103/PhysRevE.56.680.

[ref40] Gonzaleztello P.; Camacho F.; Blazquez G. Density and viscosity of concentrated aqueous-solutions of polyethylene-glycol. J. Chem. Eng. Data 1994, 39, 611–614. 10.1021/je00015a050.

[ref41] Bittrich E.; Kuntzsch M.; Eichhorn K.-J.; Uhlmann P. Complex pH- and temperature-sensitive swelling behavior of mixed polymer brushes. J. Polym. Sci., Part B: Polym. Phys. 2010, 48, 1606–1615. 10.1002/polb.22021.

[ref42] Chu L.-Y.; Li Y.; Zhu J.-H.; Chen W.-M. Negatively thermoresponsive membranes with functional gates driven by zipper-type hydrogen-bonding interactions. Angew. Chem., Int. Ed. 2005, 44, 2124–2127. 10.1002/anie.200462687. 15736236

[ref43] Lutkenhaus J. L.; Hrabak K. D.; McEnnis K.; Hammond P. T. Elastomeric flexible free-standing hydrogen-bonded nanoscale assemblies. J. Am. Chem. Soc. 2005, 127, 17228–17234. 10.1021/ja053472s. 16332070

[ref44] Yu J.; Jackson N. E.; Xu X.; Morgenstern Y.; Kaufman Y.; Ruths M.; de Pablo J. J.; Tirrell M. Multivalent counterions diminish the lubricity of polyelectrolyte brushes. Science 2018, 360, 1434 10.1126/science.aar5877. 29954973

[ref45] Kuhl T. L.; Leckband D. E.; Lasic D. D.; Israelachvili J. N. Modulation of interaction forces between bilayers exposing short-chained ethylene-oxide headgroups. Biophys. J. 1994, 66, 1479–1488. 10.1016/S0006-3495(94)80938-5. 8061197 PMC1275868

[ref46] de Gennes P. G. Polymers at an interface - a simplified view. Adv. Colloid Interface Sci. 1987, 27, 189–209. 10.1016/0001-8686(87)85003-0.

[ref47] Israelachvili J. N. Intermolecular and Surface Forces; 3rd ed.; Academic Press: Burlington, MA, 2011; 674 pp.

[ref48] Schwenzfeier K. A.; Erbe A.; Bilotto P.; Lengauer M.; Merola C.; Cheng H.-W.; Mears L. L. E.; Valtiner M. Optimizing multiple beam interferometry in the surface forces apparatus: Novel optics, reflection mode modeling, metal layer thicknesses, birefringence, and rotation of anisotropic layers. Rev. Sci. Instrum. 2019, 90, 043908 10.1063/1.5085210. 31043001

[ref49] Satulovsky J.; Carignano M. A.; Szleifer I. Kinetic and thermodynamic control of protein adsorption. Proc. Natl. Acad. Sci. U. S. A. 2000, 97, 9037–9041. 10.1073/pnas.150236197. 10908651 PMC16817

[ref50] Paraskevopoulou V.; Falcone F. H. Polyionic tags as enhancers of protein solubility in recombinant protein expression. Microorganisms 2018, 6, 47 10.3390/microorganisms6020047. PMC6027335 29882886

