# Supplementary material for: Control of Polymer Brush Morphology, Rheology, and Protein Repulsion by Hydrogen Bond Complexation
Source: Langmuir. 2021 Apr 14;37(16):4943–52. doi: 10.1021/acs.langmuir.1c00271 (PMC8154870; doi:10.1021/acs.langmuir.1c00271)
Supplement: Supplementary file 1 — la1c00271_si_001.pdf [file la1c00271_si_001.pdf]

Control of polymer brush morphology, rheology and protein repulsion by hydrogen bond complexation

John Andersson,[1] Gustav Ferrand-Drake del Castillo,[1] Pierluigi Bilotto,[2] Fredrik Höök,[3] Markus Valtiner[2] and Andreas Dahlin.[1]

1 Department of Chemistry and Chemical Engineering, Chalmers University of Technology, 41296 Gothenburg, Sweden.

2 Institute of Applied Physics, Group of Applied Interface Physics, Vienna University of Technology, 1040 Vienna, Austria.

3 Department of Physics, Chalmers University of Technology, 41296 Gothenburg, Sweden.

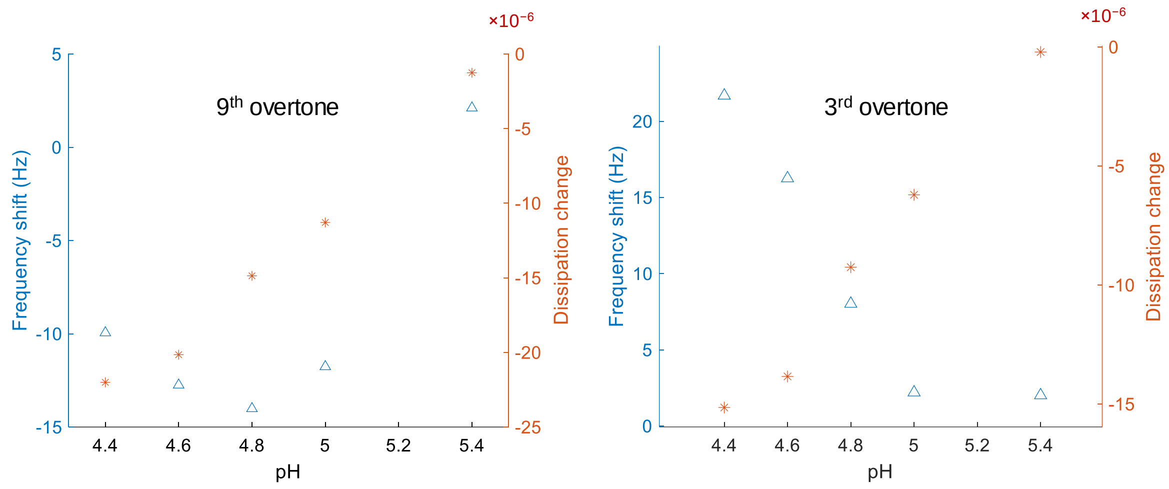

Figure S1 Example of QCMD data for estimating the pH_{crit} of about 5.2 (using 75 kg/mol PMAA). Saturated signals after exposing PEG to PMAA are plotted. At the 9th overtone, significant signals are observed at pH 5.0 but not 5.4. At the third overtone (which exhibits positive signals), the dissipation change is noticeable when going down to pH 5.0 compared to 5.4.

Estimating the number of hydrogen bonds

Polyacids do not follow simple (Henderson–Hasselbalch) titration behavior like monovalent carboxylic acids due to self-repulsion and counterions attracted to the partly charged chains. In other words, the acidic groups are not independent of each other and the equilibrium constant depends on the degree of ionization. (This is essentially an extension of the well-known multiple pK_a values for acids such as oxalic acid, citric acid etc.)

One model for polyelectrolytes assumes that the pH of the surrounding liquid bulk influences the fraction of ionized groups f according to:[1]

$$\mathrm{pH} = \mathrm{pK_0} + \log_{10}\left(\frac{f}{1-f}\right) + A \times f^{1/3} \tag{S1}$$

Here pK_0 represents the pK_a in the limit of $f = 0$ and A is a constant depending on the surrounding environment, in particular ionic strength. Experimental titrations have shown that for poly(acrylic acid), this model is valid for any degree of protonation, while for PMAA, the model holds as long as $f < 0.2$ with pK_0 = 4.28 and A = 2.64 close to physiological ionic strength.[1] From our experiments we could identify quite precisely the critical pH (pH$_{crit}$) below which binding started to occur. Following Equation S1, one can thus identify the corresponding number of hydrogen bond donors (n_{crit}) at this pH:

$$\mathrm{pH_{crit}} = 4.28 + \log_{10}\left(\frac{N}{n_{crit}} - 1\right) + 2.64\left[1 - \frac{n_{crit}}{N}\right]^{1/3} \tag{S2}$$

Here N is the degree of polymerization. We use the the smallest PMAA (M_n = 1.3 kg/mol) with N = 15 monomers on average to make an estimate. We identified a lower pH$_{crit}$ of about 4.5 for this molecular weight of PMAA (compared to 5.2 for the larger ones). Using Equation S2 gives an n_{crit} between 13 and 14 (and f is ~10%, i.e. low enough for the model to be valid). However, the unavoidable polydispersity makes this an underestimation: there are quite many chains with many more monomers and it may be these that bind to the surface. An upper estimate of n_{crit} is given by the fact that chains with more than $N \approx 50$ monomers are very rare (Fig. S2). This translates to n_{crit} = 45 (for the same pH$_{crit}$ = 4.5). Thus we conclude that n_{crit} should be somewhere in the range 10-50.

[1] Arnold *J. Coll. Sci.* **1957**, 12, 549-556.

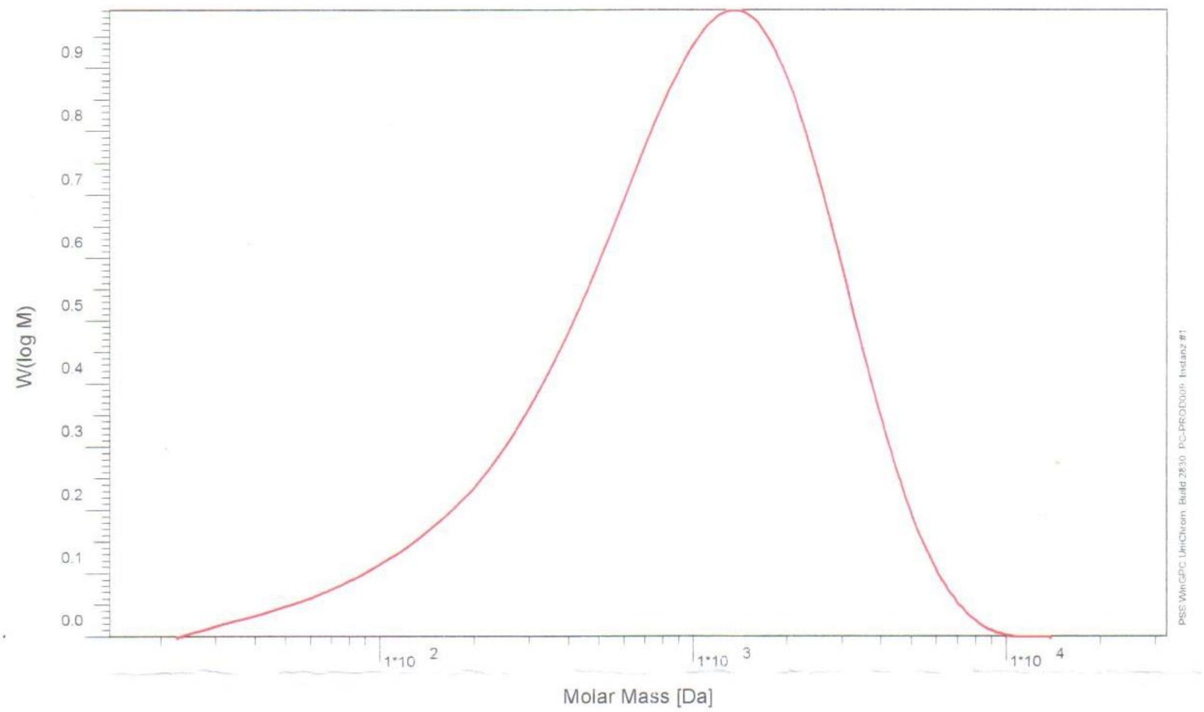

Figure S2 Polydispersity of the smallest PMAA (copied from the certificate of analysis from the supplier), for which a lower pH_{crit} of 4.5 was identified.

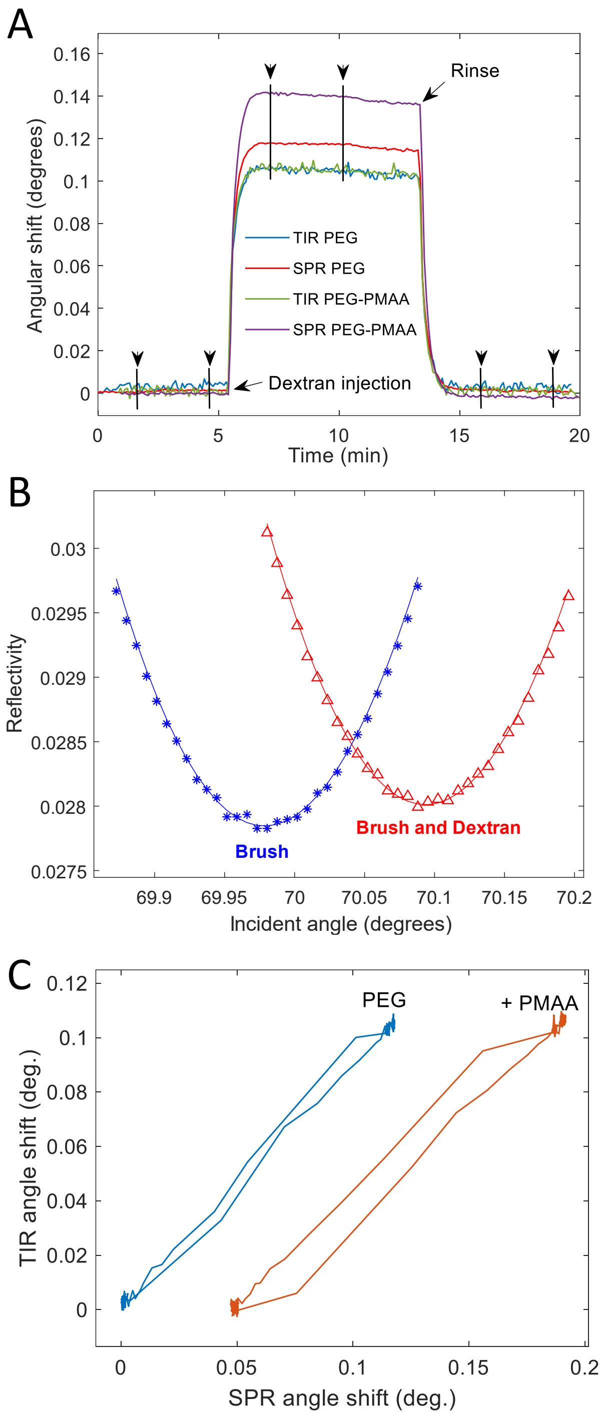

Figure S3 (A) Example of SPR and TIR angles time trace during dextran injections for probing exclusion height. The arrows indicate regions from which an average spectra is calculated in the presence or absence of the probe. (B) Example fits around the reflectivity minimum with and without probe (PEG brush without PMAA), yielding a unique refractive index and thickness for the brush when it is treated as a homogenous film. (C) Plots of TIR angle vs SPR angle during dextran injections (red curve is offset to the right by 0.05°).

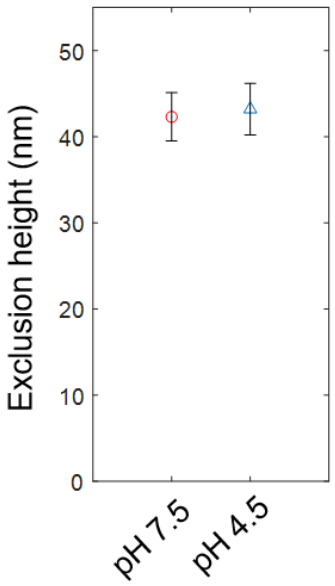

Figure S4 Control experiment showing exclusion height of PEG brushes measured at different pH with dextran as probe. The heights are the same since there is no pH-responsive component present.

PMAA adsorption on gold

Both SPR and QCMD was used to investigate PMAA interacting with a clean gold surface. SPR data (Fig. S5A) showed that PMAA (at pH 4.5) adsorbs irreversibly. After raising the pH to 7.5 or 11, the polymer remains. The shifts occurring from the buffer changes between high and low pH are about 7 times higher when PMAA is bound to the surface compared the same buffer change on an empty gold sensor. This increased shift corresponds to the ionization of the acidic groups[2] (see also main text). Similarly, Fig. S5B shows irreversible PMAA binding to gold monitored in QCMD. The signals are low, which indicates that PMAA adsorbs rigidly and adopts a flat conformation. Upon raising the pH, overtone-dependent changes in frequency and dissipation occur mainly because of increased hydration. As a control, these changes are much smaller before PMAA has been bound (first minutes in the data trace). This confirms that the polymer remains on gold even after high pH rinsing and suggests a very thin layer in direct contact with gold.

[2] Ferrand-Drake del Castillo et al. *J. Phys. Chem. C* **2018**, 122, 27516-27527.

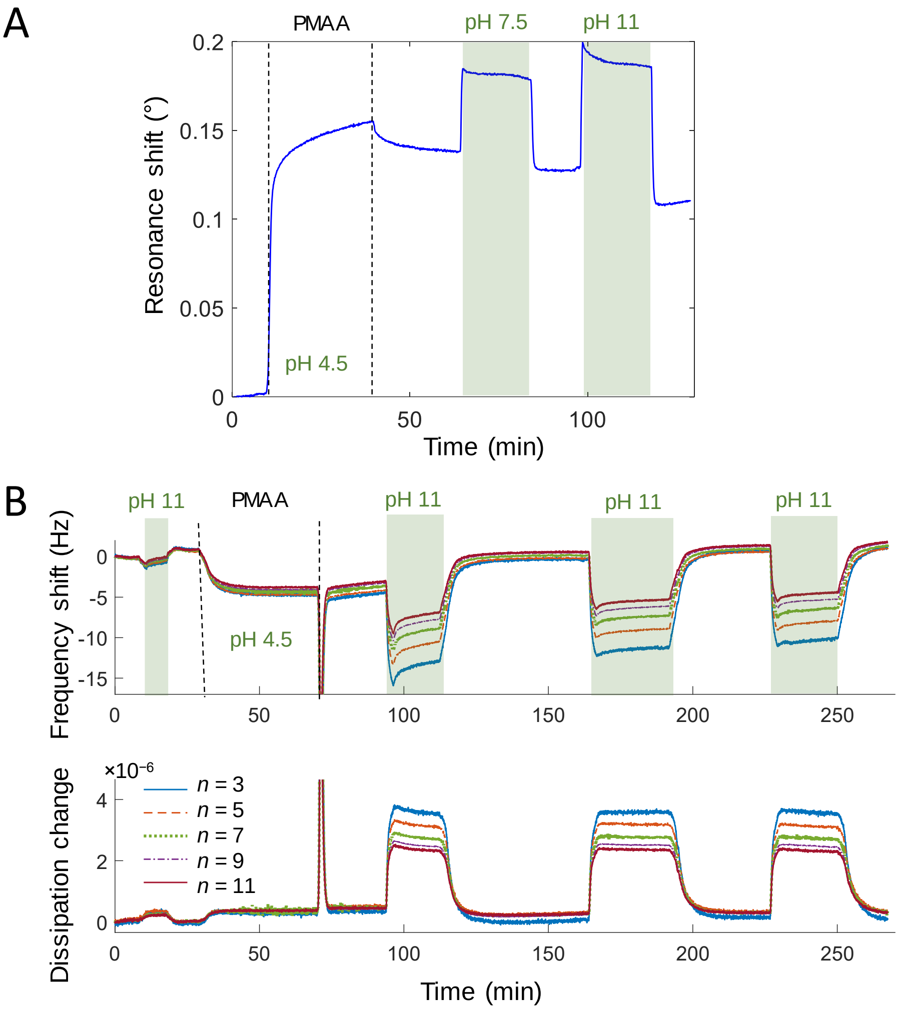

Figure S5 (A) SPR measurement at 785 nm during injection of PMAA at pH 4.5 on gold and of PBS solutions at higher pH. PBS pH 4.5 was used as running buffer. (B) QCMD measurement during injection of PMAA at pH 4.5 on gold and injections of PBS with pH increased to 11.

Molar refractivity

The value for the molar refractivity of PEG was taken from our previous work.[3] The molar refractivity of PMAA was determined using the TIR angle shift in SPR upon injecting different concentrations (Fig. S6). Note that this value is relatively low and that PMAA (like other polyelectrolytes) will have a higher molar refractivity at physiological pH where it is charged.

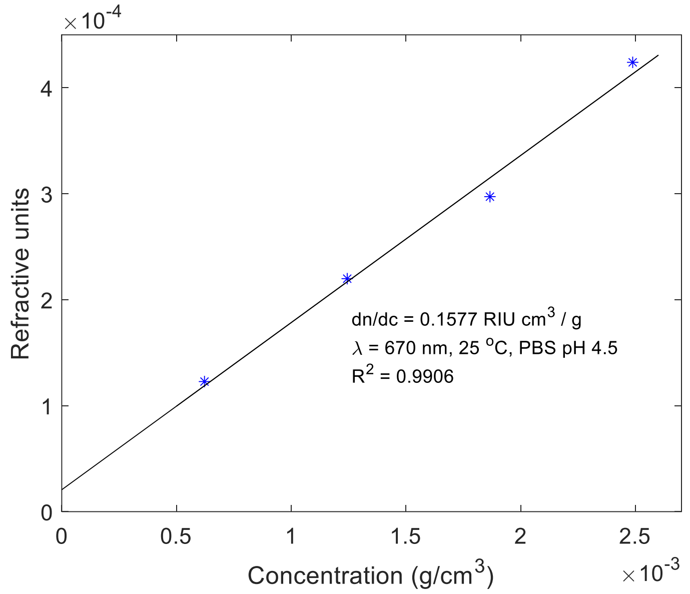

Figure S6 Molar refractivity of PMAA at pH 4.5 determined from the refractive index at different mass concentrations (75 kg/mol). The refractive index values were obtained directly from the TIR angle in the SPR instrument using Snell's law.

[3] Emilsson et al. *ACS App. Mater. Inter.* **2015**, 7, 7505-7515.

Viscoelastic model

To model the rheology of the brush using QCMD, the initial absolute frequency and dissipation were measured in the same liquid environment. Fig. S7 shows the same data as in the main text, but signals are vs the clean surface. In other words, the starting values correspond to the signals from the PEG brush. Using the simplest Voigt model, the film is described by four parameters which are assumed to be independent of frequency: density (ρ), thickness (h), shear modulus (μ) and shear viscosity (η). For a film that is thinner than the acoustic wave extension into the (Newtonian) liquid above, the frequency and dissipation shifts can be approximated by a linearization as:[4]

$$\Delta f_n \approx -\frac{1}{2\pi\rho_0 h_0}\left(\frac{\eta_{\mathrm{bulk}}}{\delta_{\mathrm{bulk}}} + 2\pi h\rho f_n - 2h\left[\frac{\eta_{\mathrm{bulk}}}{\delta_{\mathrm{bulk}}}\right]^2 \frac{\eta[2\pi f_n]^2}{\mu^2+[2\pi\eta f_n]^2}\right) \quad \text{(S3)}$$

$$\Delta D_n \approx -\frac{1}{2\pi f_n\rho_0 h_0}\left(\frac{\eta_{\mathrm{bulk}}}{\delta_{\mathrm{bulk}}} + 2h\left[\frac{\eta_{\mathrm{bulk}}}{\delta_{\mathrm{bulk}}}\right]^2 \frac{2\pi\mu f_n}{\mu^2+[2\pi\eta f_n]^2}\right) \quad \text{(S4)}$$

Here ρ_0 and h_0 are density and thickness for the quartz oscillator and η_{bulk} is the viscosity of the bulk liquid. The overtone (n) dependence comes in through the different resonance frequencies f_n. The δ parameter is the penetration depth of acoustic waves in the medium defined as:

$$\delta = \sqrt{\frac{\eta_{\mathrm{bulk}}}{\pi\rho_{\mathrm{bulk}} f_n}} \quad \text{(S5)}$$

[4] Voinova et al. *Physica Scripta* **1999**, 59, 391-396.

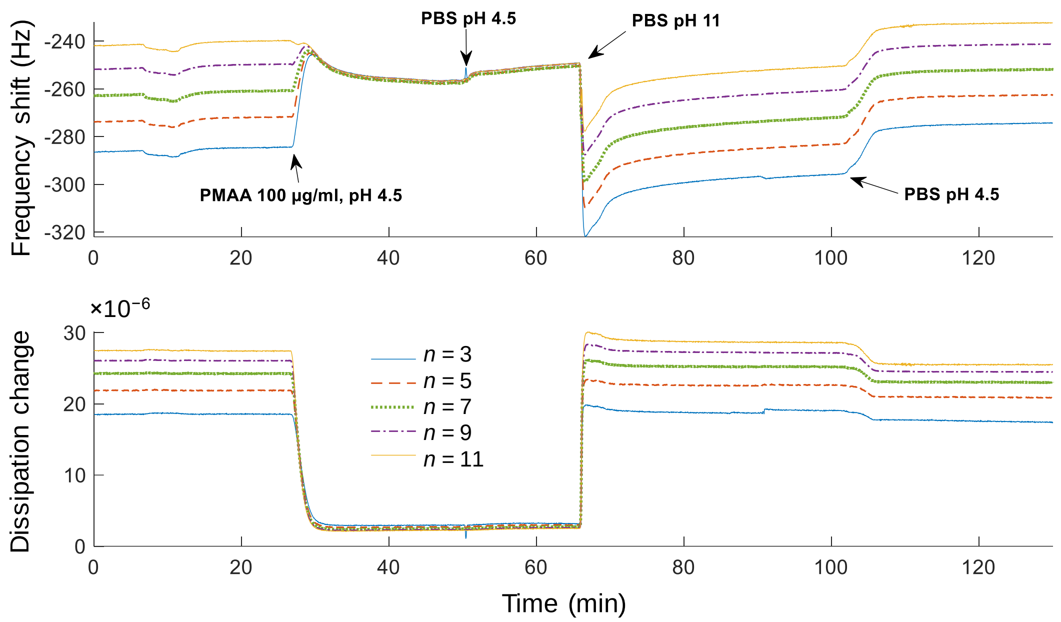

Figure S7 QCMD data presented in absolute signals compared to a bare sensor crystal (same data as in main text). The values at time zero represent the PEG brush in PBS.

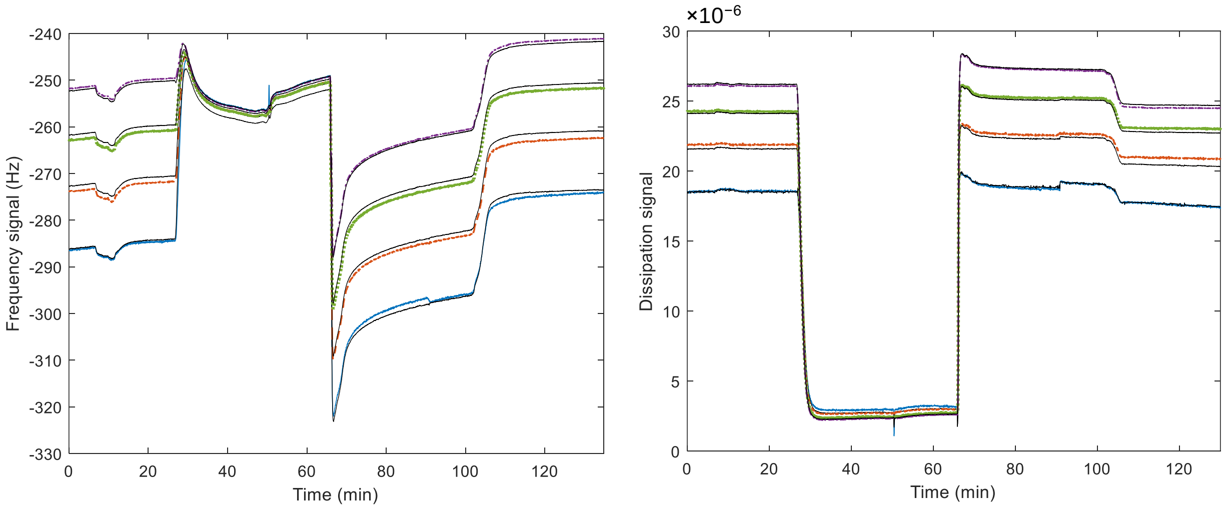

Figure S8 Fit for the Voigt model (black lines). Overtone 11 was not included.

Supplementary SFA data

Fig. S9 presents the FECO observable while performing a SFA experiment. We have on the x-axis the wavelength and on the y-axis the intensity of light. The wavelength of maximum intensity is a function of the distance between two semi-reflecting mirrors that define the optical cavity and the refracting index of the medium confined inside. Only specific wavelengths can escape the optical cavity depending on the distance between the mirrors, generating the FECO. The emission spectrum of a reference light (Hg lamp) allows identification of the light spectrum region (green and yellow lines on the right of Fig. S9). Using the multiple matrix method[5] we fitted the spectrum while letting the distance between the two semi-reflecting mirrors vary.

First, we performed a simple contact experiment in air for a clean gold surface and a back silvered mica (part A), resulting in the reference point corresponding to the mica thickness H_{mica}. A new element in the gold-mica-silver cavity can be modelled as a homogenous film with a specific refractive index and thickness. We grafted a PEG layer on the gold surface and performed again the SFA contact measurement in air. As indicated by the red line (part B), the FECO of the new system is red-shifted. Assuming a refractive index of 1.456 for the dry PEG (same as in the SPR analysis), this shift in wavelength is turned into a thickness, resulting in $H_{\text{PEG}} = 9.75 \pm 0.1$ nm.

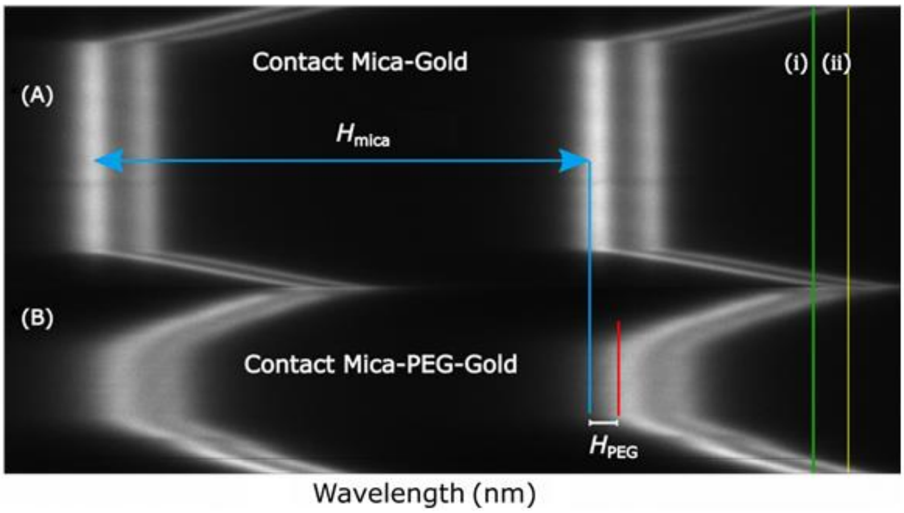

Figure S9 FECO in dry state. (A) Contact between a gold surface and a back silvered mica surface. By the displacement in wavelength (double blue arrow) one can define the thickness of the mica sample H_{mica} which will define the pseudo zero of the system. (B) When a PEG layer is added on the gold substrate, the FECO in contact will present a red shift (red line) which is used to calculate the layer thickness H_{PEG}.

[5] Schwenzfeier et al. *Rev. Sci. Instrum.* **2019**, 90, 043908.
